# Mr. Paget’s Report Concluded

**Published:** 1844-06-01

**Authors:** 


### MR. PAGET'S REPORT CONCLUDED.

*Age of puberty in girls.* Mr. Roberton of Manchester, in continuation of some former papers, the object of which was to prove that the age of puberty is as early in the cold as in the tropical regions of the earth, and that the early fecundity in Hindostan and other warm countries is only the consequence of early marriages, proceeds now to show, that in all countries alike, early marriages (and early fecundity) are always connected with moral and political degradation, as exhibited in bad laws and customs, the enslavement more or less of the women, ignorance of letters, impure and debasing systems of religion; and that they bear no relation to the climate of the country.

His evidence is extensive and very interesting; and the conclusions he arrives at are, 1. That in England, Germany, and Protestant Europe in general, early marriage, i. e. marriage about the age of puberty, is comparatively rare. 2. That early marriage prevails among the uncivilized tribes within the arctic circle, as it likewise does in *all* cold countries, the inhabitants of which are in a state of ignorance and moral degradation. 3. That throughout European Russia, which is confessedly low in civilization, extremely premature marriage was the universal custom at no distant date. 4. That at the present day, in the most southerly countries of Europe, where the people are immersed in superstition and ignorance, marriage is early. 5. That in Ireland, which as to its moral condition somewhat resembles the last-mentioned countries, the marriage union takes place among the Roman Catholic population almost as early. 6. That in England, about two centuries ago, when debasing political and social circumstances combined to favour the practice, early marriages were general, at all events in the upper ranks. 7. That in all the countries to which reference has been made, juvenile marriage is invariably seen as an attendant upon ignorance and moral debasement, and this without reference to *climate.* 8. That consequently it is allowable to infer that early marriage in oriental countries (which has generally, but without any proof, been ascribed to precocious puberty,) depends solely on the same moral and political causes as produce it elsewhere; more especially as those very causes are well known to exist at present in an aggravated degree in all oriental and intertropical countries.

These conclusions are probably in a great measure true; yet that the commencement of menstruation and of fecundity does bear some relation to the latitude and average temperature, appears to be proved by the following table, in which M. Raciborski gives his results as to the average age at which menstruation commences in different countries and towns:

| Name of Town. | Latitude. | Age at first menstruation. | Mid. Temp. of the year. | No. of Observations. | Observers. |
|---|---|---|---|---|---|
| Toulon | 43° | 14·081 | 15° | 43 | Marc d'Espene. |
| Marseilles | 43 | 14·015 | 15· | 25 | Ditto. |
| Lyons | 46 | 14·492 | 11·6 | 160 | Bouchacourt. |
| Paris | 49 | 14·465 | 10·6 | 200 | Raciborski. |
| Gottingen | 52 | 16·038 | 8· | 137 | Osiander. |
| Warsaw | 52 | 15·083 | 9·2 | 100 | Lebrun. |
| Manchester | 53 | 15·191 | 9·6 | 450 | Roberton. |
| Skeen | 59 | 15.450 | 6· | 100 | Faye. |
| Stockholm | 29 | 15.590 | 5.7 | 102 | Wistrand. |
| Swedish Lapland | 65 | 18· | 4· | | Wretholm. |

In general, therefore, the period of puberty is later in nearly the same ratio as the latitude is higher: for each degree of the one the other is retarded about a month and a few days. And the lower the latitude, the more frequent are the examples of precocious appearance of menstruation.

A still more exact relation is between the date of first menstruation and the mean year's temperature; as may be seen by comparing Warsaw and Göttingen, Göttingen and Manchester, &c. M. Raciborski adds that *race* often determines the period of first menstruation. The children of negroes born in England menstruate as early as their parents; those of Europeans born in India as late as their parents. To determine how far circumstances of climate could countervail the influence of race, M. Raciborski obtained information respecting the period of menstruation in Jewesses in Poland, from M. Lebrun, médecin-en-chef of a hospital in Warsaw, and found the mean period in Catholics 15·83, in Jewesses 15·89; (100 observations of each race;) showing that the influence of race remained after ten or more centuries. And in like manner the menstruation ceases sooner in Polish Jewesses than in Sclavonian women, lasting in the former on an average $29\frac{28}{33}$ years, and in the latter $31\frac{6}{33}$ years.

There is a difference also, dependent, probably, on numerous causes, between the women of Paris and those of villages a league and half or two leagues from Paris, though both have a similar soil, temperature, &c. In the villages the average age at first menstruating is 15·020 years, in Paris 14·465.

M. Raciborski has also published an account of the age at which menstruation ceases. At Lyons the average age is between 45 and 50; at the Salpêtrière, in 100 women, the average was 46·03; at Warsaw, 47·05; at Christiana, 48·07. As a general rule, the greater the number of children borne, the longer is the continuation of menstruation; and the earlier the commencement of menstruation, the greater the number of children and the later the cessation.

*Changes in age—Varia.* A remarkable example of vigour in most advanced age has been observed in M. Rochard, a musician, 107 years old, on whom Mr. Cæsar Hawkins successfully operated for strangulated hernia. The hernia had been strangulated upwards of thirty hours: the wound united by the first intention, except where two ligatures hung out, and in a fortnight after the operation the old man was as well as before it. He has since died.

M. Ruelle, of Cambrai, has recorded an example of precocious virility. A child, three years and a quarter old, muscular and strong as one of eight, has all his male organs of the full adult size, with long black hair on the pubes, and under excitement discharges semen four or five times daily. He has also a full male voice, and dark short hair on the cheek and upper lip.

A contribution to the knowledge of the effects of the air at great heights in the atmosphere, and a confirmation of most of the results which have been already obtained are furnished in the observations made in an account of an ascent of the 'Grosse Venediger,' a mountain upwards of 11,000 Austrian feet high, in the southern border of Oberpinzgan, by Dr. F. Spitaler. The party ascending consisted of forty persons, of whom twenty-six only accomplished the feat. The chief effects produced were, 1. On the respiration, which in all became rapid and difficult, and was greatly hurried in exertion, and in some amounted to agony, so that from mere dyspnœa they were compelled to return: one also had slight hemoptysis. 2. On the pulse, which became small and weak. 3. On the secretion of urine, which was remarkably diminished, so that among the whole forty persons, during between eight and nine hours' walking in a temperature only just above freezing point, urine was passed only nine times. 4. On the cutaneous exhalation, which (though the invisible evaporation was probably much increased,) did not once appear as sweat. 5. On the heat of the body. All had the sensation of intolerable cold, though actively exerting themselves and well clothed, and though the temperature was 4° or 5° R., and the weather nearly calm. This was felt, however, only when they had ascended above 9000 feet: below it, although the temperature was not higher, the sensation of cold was less painful. 6. On the muscular power, which was, in all the party after they had nearly attained the height of 1000 feet, exceedingly prostrated. Some were from utter fatigue obliged to give in, some could not even stand, and of those who went on none could walk more than twenty or, at last, more than ten steps without stopping to rest. In some these signs were accompanied by ringing in the ears, in some by nausea and even by vomiting, in some by utter carelessness of life; not one reached the summit except in a state of complete exhaustion. And all this was far from being the kind of fatigue consequent on extraordinary muscular exertion; for several of those who could not attain the summit, and of those who did so only with difficulty, descended in good plight, and walked on for many hours with scarcely a complaint of weariness.

The influence of the increased brightness of the sun's light was very marked. The clear, deep blue of the most beautiful southern sky was far surpassed in beauty; and though all the party wore dark shades or vails, all suffered from pain and inflammation of the face and eyes, and of every part which was at all exposed to the action of the sun.

Dr. D. D. Owen has given a detailed account of the impressions of two human feet found on the surface of a slab of limestone, from the specimen described by Mr. Schoolcraft in 1822, and by Dr. Mantell in his 'Wonders of Geology.' The slab of limestone was taken from a rock which was exposed at the very margin of the Mississippi, opposite St. Louis, but only during very low water, such as does not happen more than once in ten years. It is a soiled mass, upwards of a ton weight, of a purple and grayish tint, containing numerous shells, species of producta and pentromytes. The impressions are so exactly like those of feet set upon a soft mud, that it is difficult to imagine that they can be a work of art; yet Dr. Owen thinks the difficulties of this hypothesis less than those of that which ascribes to them an existence before the limestone had hardened.

